# LONG-TERM REPEATED BOTULINUM TOXIN A TREATMENT OVER 12 YEARS GRADUALLY CHANGES GAIT CHARACTERISTICS: SINGLE-CASE STUDY

**DOI:** 10.2340/jrmcc.v7.40827

**Published:** 2024-09-03

**Authors:** Hiroki TANIKAWA, Hitoshi KAGAYA, Shota ITOH, Kento KATAGIRI, Hikaru KONDOH, Kenta FUJIMURA, Satoshi HIRANO, Toshio TERANISHI

**Affiliations:** 1 Faculty of Rehabilitation, School of Health Sciences, Fujita Health University, Toyoake, Aichi, Japan; 2 Department of Rehabilitation Medicine, National Center for Geriatrics and Gerontology, Obu, Aichi, Japan; 3 Department of Rehabilitation Medicine, School of Medicine, Fujita Health University, Toyoake, Aichi, Japan; 4 Department of Rehabilitation, Fujita Health University Hospital, Toyoake, Aichi, Japan

**Keywords:** muscle spasticity, gait analysis, gait disorders, botulinum toxins, long-term treatment

## Abstract

**Objective:**

To demonstrate the long-term efficacy of repeated botulinum toxin A injections into the same muscles for ameliorating lower limb spasticity and gait function.

**Design:**

Single-case study

**Patient:**

A 36-year-old woman with right cerebral haemorrhage received her first botulinum toxin A injection 1,296 days after onset. The patient underwent 30 treatments over 12 years after the first injection to improve upper and lower limb spasticity and abnormal gait patterns. The mean duration between injections was 147 days.

**Methods:**

The Modified Ashworth Scale, passive range of motion, gait velocity, and degree of abnormal gait patterns during treadmill gait were evaluated pre-injection and at 2, 6, and 12 weeks after every injection.

**Results:**

The follow-up period showed no injection-related adverse events. Comfortable overground gait velocity gradually improved over 30 injections. The Modified Ashworth Scale and passive range of motion improved after each injection. Pre-injection values of the degree of pes varus, circumduction, hip hiking, and knee extensor thrust improved gradually. However, the degree of contralateral vaulting, excessive lateral shift of the trunk, and insufficient knee flexion did not improve after 30 injections.

**Conclusion:**

Repeated botulinum toxin A injections effectively improve abnormal gait patterns, even when a single injection cannot change these values.

## Lay Abstract

Botulinum toxin A (BoNTA) is used to treat spasticity in the arms and legs of adult patients. We report a case of a woman who was treated with BoNTA 30 times over 12 years to improve limb spasticity and abnormal gait patterns. The range of motion improved after each injection, and some other features of her gait improved gradually. We did not observe any injection-related adverse events during the follow-up period. We conclude that repeated BoNTA injections can effectively improve some abnormal gait patterns and comfortable overground gait velocity, even when a single injection cannot change these values. Cumulative effects were also shown. The combination of BoNTA and rehabilitation may lead to better results.

Botulinum toxin A (BoNTA) treatment for upper and lower limb spasticity in adult patients has been covered by medical insurance since 2010 in Japan. Over the years, BoNTA injections have been widely used to reduce spasticity in patients recovering from stroke. A meta-analysis reported that BoNTA injections significantly reduced lower limb spasticity (1), and our previous studies have demonstrated their beneficial effects on gait velocity and abnormal gait patterns (2, 3). At present, many patients with stroke undergo BoNTA treatment repeatedly; therefore, our clinical interests revolve around the following 2 questions:

1. Do 1-time injection and long-term multiple-time injections have the same effect?
2. Are the effects of repeated BoNTA injections on lower limb function cumulative?

Previous studies have indicated that the effect of multiple time BoNTA injections was the same as that of first-time injection; however, injections in these studies were carried out <10 times (4–7). Recently, many patients have undergone BoNTA injections >10 times. Although it is necessary to demonstrate the effects of long-term repeated BoNTA injections, we understand the difficulty in collecting such data of patients who underwent multiple BoNTA injections at the same dose for the same muscles in clinical settings.

Therefore, we report the case of a patient who received BoNTA injections 30 times over 12 years after the first BoNTA injection. We aimed to demonstrate the efficacy of long-term repeated BoNTA into the same muscles on lower limb spasticity and gait function.

## CASE REPORT

A 36-year-old woman (height, 168.0 cm; weight, 51.0 kg) with right cerebral haemorrhage received a BOTOX (GlaxoSmith-Kline,Tokyo, Japan) injection for the first time 1,296 days after the onset of haemorrhage to reduce spasticity and improve abnormal gait patterns. Before the first injection, the patient displayed a moderate degree of paralysis (the Stroke Impairment Assessment Set-Motor Function [SIAS-M] score was 3-1B, 4-3-1) and could take care of herself and walk without orthosis; however, she exhibited abnormal gait patterns such as circumduction, hip hiking, knee extensor thrust, contralateral vaulting, excessive lateral shift of the trunk over the unaffected side, and insufficient knee flexion during the swing phase. The patient showed upper and lower limb spasticity and wanted treatment for pes varus during the swing phase in gait. An experienced physiatrist evaluated each muscle’s tone, observed the patient’s gait, selected the target muscle, and determined the BoNTA dose. BOTOX was diluted in saline to a concentration of 12.5–25.0 U/mL. Our institutional review board approved this clinical case report, and written informed consent was obtained from the patient.

The patient underwent treatment 30 times over the 12 years after the first BoNTA injection. The injected muscles and doses are listed in Table I. The mean ± standard deviation (SD) duration between injections was 147±19 days. In Japan, the maximum number of BoNTA injections in the upper and lower limbs on a single occasion was limited to 360–400 units during the study period. The patient did not receive physical therapy before or after the injection.

**Table I:** Botulinum toxin A doses (units)

Number of injections	Lower limb		Upper limb							
	Tibialis posterior	Flexor digitorum longus	Biceps brachii	Brachialis	Triceps brachii	Flexor carpi radialis	Flexor carpi ulnaris	Flexor digitorum superficialis	Flexor digitorum profundus	Flexor pollicis longus
1	50	-	50	-	-	50	50	50	-	50
2	75	-	75	-	-	40	30	50	-	30
3	50	50	75	-	-	40	30	50	-	30
4	50	50	75	-	-	40	30	50	-	30
5	50	50	75	-	-	25	25	50	-	25
6	50	50	75	-	-	25	25	20	-	25
7	50	50	75	-	-	25	25	20	-	25
8	50	50	50	25	-	25	25	50	-	25
9	50	50	50	25	-	25	25	50	-	25
10	50	50	50	25	-	25	25	50	-	25
11	50	50	50	25	-	25	25	50	-	25
12	50	50	50	25	-	25	25	50	-	25
13	50	50	50	25	-	25	25	50	-	25
14	50	50	50	25	-	25	25	50	-	25
15	50	50	50	25	-	25	25	50	-	25
16	50	50	50	25	-	25	25	50	-	25
17	50	50	50	25	-	25	25	50	-	25
18	50	50	50	25	-	25	25	50	-	25
19	50	50	50	25	-	25	25	25	-	25
20	50	50	50	25	-	25	25	50	-	25
21	50	50	50	25	-	25	25	50	-	25
22	50	50	50	25	-	25	25	25	-	25
23	50	50	50	-	25	25	25	50	-	25
24	50	50	50	-	-	25	25	-	50	25
25	50	50	50	25	-	25	25	50	-	25
26	50	50	50	25	-	25	25	50	-	25
27	50	50	50	25	-	25	25	50	-	25
28	50	50	50	25	-	25	25	50	-	25
29	50	50	50	25	-	25	25	50	-	25
30	50	50	50	25	-	25	25	50	-	25

The Modified Ashworth Scale (MAS) score of the ankle flexors, passive range of motion (ROM) of ankle dorsiflexion, comfortable overground gait velocity, pes varus angle, and degree of abnormal gait patterns during treadmill gait (Tables II and III) (8–11) were evaluated just before the injection and at 2, 6, and 12 weeks after every injection. A comfortable overground gait velocity was evaluated on a 12-m-long walkway without an orthosis. The mean walking velocity through the central 10 m of the walkway was obtained from 2 trials. The pes varus angle and degree of abnormal gait patterns during treadmill gait were calculated using a 3-dimensional motion analysis system (KinemaTracer; KISSEI CMOTEC, Matsumoto, Japan). The validity of the calculation of the pes varus angle and the indices of abnormal gait patterns using this system have been verified in previous studies (2, 8–11). The treadmill speed was set to a comfortable pre-injection overground gait velocity for each injection.

**Table II:** Definitions for abnormal gait patterns (8–11)

Abnormal gait pattern	Definition
Circumduction gait	The lower extremity of the affected side shows hip joint abduction and lateral rotation during the initial to mid-swing and hip joint adduction and medial rotation during the mid-swing to terminal swing, following a semicircular trajectory.
Hip hiking	The pelvis on the affected side is raised during pre-swing to mid-swing, associated with shortening of the trunk on the affected side.
Contralateral vaulting	The excessive pelvic rise by the unaffected side knee extension during the stance phase of the unaffected side.
Knee extensor thrust	A dynamic, rapid knee extension during the loading response to the terminal stance of the affected leg.
Insufficient knee flexion during the swing phase	A decreased maximum knee flexion angle during the swing phase of the affected leg.
Excessive lateral shift of the trunk over the unaffected side	The trunk shifts excessively over the unaffected side during the swing phase of the affected leg.

**Table III:** Calculation of index values for abnormal gait patterns (8–11)

Abnormal gait pattern	Definition
Circumduction gait	The difference in distance between the lateral-most X coordinate of the ankle joint marker in 25–75% of the swing phase and the medial-most X coordinate in 25–75% of the stance phase, corrected by lower limb length.^a^
Hip hiking	The difference between the maximum value of the Z coordinate of the hip joint marker during the swing phase and the Z coordinate of the contralateral hip joint marker at the same time corrected for the mean left-right difference of the Z coordinate during the double-support phase.
Contralateral vaulting	The difference between 2 distances while in the portion of the double-stance phase in which the affected leg is located behind the unaffected leg, corrected by the stride length. One distance is between the Z coordinate of the maximum and minimum values of the affected hip joint. The other distance is between the Z coordinates of the maximum and minimum values of the affected ankle joint.
Knee extensor thrust	The difference between the maximum Y coordinate velocity of the knee of the affected leg in the single stance phase and the treadmill gait speed.
Insufficient knee flexion during the swing phase	The percentage of the maximum knee flexion angle during the swing phase compared to the angle of knee flexion in healthy individuals.
Excessive lateral shift of the trunk over the unaffected side	The average distance between (1) the lateral-most X coordinate of the midpoint between the bilateral acromions in the part of the double-stance phase where the affected leg is located behind the unaffected leg and the swing phase of the affected leg and (2) the average X coordinate of the midpoint between the bilateral ankle joints in the part of the double stance phase in which the affected leg is located behind the unaffected leg, corrected by lower limb length.^a^

a Corrected by lower limb length: the index value was expressed as a percent of the Z coordinate of the hip joint in a quiet standing position.

No injection-related adverse events were observed, and the degree of paralysis according to the SIAS-M score did not change through the follow-up period. Missing data were observed in 3.3% (4/120 times of evaluation) due to the patients’ condition.

**Fig. 1:**
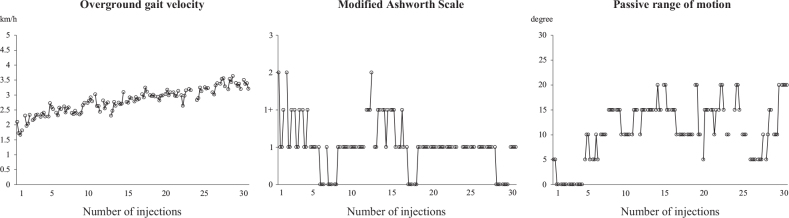
Comfortable overground gait velocity, Modified Ashworth Scale (MAS) score of ankle flexors, and passive range of motion (ROM) of ankle dorsiflexion before and after all injections. Each evaluation score was plotted. The patient was evaluated 4 times at each injection (just before the injection and at 2, 6, and 12 weeks after the injection), and the 4 values were connected. The X-axis indicates the number of injections.

**Fig. 2:**
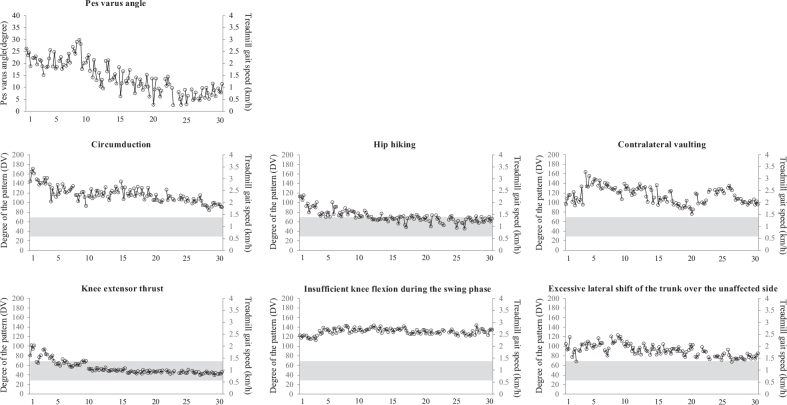
Abnormal gait patterns before and after all injections. The maximum pes varus angle during the swing phase, degree of abnormal gait patterns, and treadmill belt speed at that time (grey) are shown. The patient was evaluated 4 times at each injection (just before the injection and at 2, 6, and 12 weeks after the injection), and the 4 values were connected. The indices for the degree of abnormal gait patterns were calculated as the deviation values from healthy individuals; deviation 50 indicates the average of healthy individuals, while deviation 30 (mean–2 SD) to 70 (mean+2SD) indicates the normal range. Using the deviation value, 1 can easily understand the relative severity compared with other abnormal gait patterns (8–11). The X-axis indicates the number of injections.

The comfortable overground gait velocity, MAS score of the ankle flexors, and passive ROM of ankle dorsi flexion are shown in Fig. 1. The comfortable overground gait velocity gradually improved through the course of 30 injections. The MAS score and passive ROM improved after each injection. The degree of abnormal gait patterns is shown in Fig. 2. The pre-injection values of the degree of pes varus, circumduction, hip hiking, and knee extensor thrust improved gradually.

## DISCUSSION

The MAS score and passive ROM improved after each injection (Fig. 1), as reported in previous studies (1, 2). The scores before each injection improved gradually, with > 20 injections possibly contributing to maintaining the improvement. Although comfortable overground gait velocity did not always improve after each injection, it showed gradual improvement throughout the 30 injections.

Regarding the abnormal gait patterns (Fig. 2), the pes varus angle improved 2 weeks after each injection, and the pre-injection value gradually improved after approximately 10 injections. Our previous study indicated that BoNTA treatment on a single occasion improved the degree of pes varus (2); however, the present study is the first to show the effect of repeated BoNTA treatment on the degree of pes varus. While the degree of circumduction, hip hiking, and knee extensor thrust did not always improve after each injection, the pre-injection values improved gradually. The degree of hip hiking and knee extensor thrust fell within the normal range when the number of injections increased. Circumduction and hip hiking are compensatory movements that achieve toe clearance (12). Pes varus is one of the most prevalent deformities associated with lower limb spasticity and causes poor toe clearance during the swing phase of gait and ankle instability during weight bearing (2, 13). This would be a reason to improve the overground gait velocity, degree of circumduction, and hip hiking. In addition, a previous study reported that the BoNTA injection in the tibialis posterior muscle improved the degree of knee extensor thrust, indicating reduction in stability caused by spasticity of the ankle plantar flexors (10).

By contrast, the degree of contralateral vaulting, excessive lateral shift of the trunk over the unaffected side, and insufficient knee flexion during the swing phase did not improve after 30 injections. The injected muscles were the distal parts of the lower limb (tibialis posterior and flexor digitorum longus). As contralateral vaulting and excessive lateral shift of the trunk over the unaffected side are compensatory movements (9, 11), this might explain why the injections did not lead to improvement in these abnormal gait patterns.

Aymard et al. (14) reported that a BoNTA muscular injection influences spinal excitability, and as a result, it influences muscle synergies by limiting co-contraction between antagonistic muscles during the transition phase from stance to swing while facilitating the same co-contraction during the transition phase from swing to stance. However, according to our clinical experience, an injection alone does not significantly change several abnormal gait patterns, so rehabilitation after BoNTA injection is necessary. Francis et al. (15) indicated the need for adequate time among patients to learn how to use their muscles that experience reduced tone due to BoNTA. Although providing rehabilitation in combination has been associated with better gait (16), our patient did not undergo any gait rehabilitation, which may have contributed to the lack of improvement in abnormal patterns and is a limitation of this study.

To the best of our knowledge, this is the first single-case study on long-term (follow-up over 12 years) repeated BoNTA treatment. Based on the MAS score, BoNTA injection reduced the muscle tone at rest and improved the passive ROM and degree of abnormal gait patterns related to the injected muscles. Repeated BoNTA injections are effective, even when a single injection cannot change these values. Conversely, injection into several muscles might not change abnormal gait patterns as a systemic compensation.

In addition, Lee et al. (17) showed the efficacy of BoNTA into arm muscles for static standing balance. However, there was no significant change in dynamic balance. The influences of BoNTA injection into upper limb to gait function are still controversial (17, 18), but there is a possibility that the BoNTA injection into upper muscles improved the abnormal gait patterns in this present study.

Furthermore, the effect of BoNTA treatment may vary according to the amount of activity and/or ageing of the muscles; therefore, it is necessary to investigate it in the future. A larger study is required in patients with the same conditions to investigate the effectiveness of long-term repeated BoNTA therapy in improving abnormal gait patterns.

In conclusion, long-term repeated BoNTA treatment, together with rehabilitation, holds promise for improving gait in patients with stroke.
